# The role of LINC00094/miR‐224‐5p (miR‐497‐5p)/Endophilin‐1 axis in Memantine mediated protective effects on blood‐brain barrier in AD microenvironment

**DOI:** 10.1111/jcmm.14214

**Published:** 2019-02-22

**Authors:** Lu Zhu, Meiqing Lin, Jun Ma, Wenjing Liu, Lili Gao, Shanshan Wei, Yixue Xue, Xiuli Shang

**Affiliations:** ^1^ Department of Neurology First Affiliated Hospital of China Medical University Shenyang China; ^2^ Department of Neurobiology School of Life Sciences, China Medical University Shenyang China; ^3^ Key Laboratory of Cell Biology Ministry of Public Health of China, China Medical University Shenyang China; ^4^ Key Laboratory of Medical Cell Biology Ministry of Education of China, China Medical University Shenyang China; ^5^ Department of Geriatrics First Affiliated Hospital of China Medical University Shenyang China

**Keywords:** Alzheimer's disease, blood‐brain barrier, LINC00094, Memantine, miR‐224‐5p/miR‐497‐5p

## Abstract

The dysfunction of the blood‐brain barrier (BBB) is one of the main pathological features of Alzheimer's disease (AD). Memantine (MEM), an *N*‐methyl‐d‐aspartate (NMDA) receptor antagonist, has been reported that been used widely for AD therapy. This study was performed to demonstrate the role of the MEM in regulating BBB permeability in AD microenvironment as well as its possible mechanisms. The present study showed that LINC00094 was dramatically increased in Abeta_1‐42_‐incubated microvascular endothelial cells (ECs) of BBB model in vitro. Besides, it was decreased in MEM‐incubated ECs. Silencing LINC00094 significantly decreased BBB permeability, meanwhile up‐regulating the expression of ZO‐1, occludin and claudin‐5. Furthermore, silencing LINC00094 enhance the effect of MEM on decreasing BBB permeability in AD microenvironment. The analysis of the mechanism demonstrated that reduction of LINC00094 inhibited Endophilin‐1 expression by up‐regulating miR‐224‐4p/miR‐497‐5p, promoted the expression of ZO‐1, occludin and claudin‐5, and ultimately alleviated BBB permeability in AD microenvironment. Taken together, the present study suggests that the MEM/LINC00094/miR‐224‐5p (miR‐497‐5p)/Endophilin‐1 axis plays a crucial role in the regulation of BBB permeability in AD microenvironment. Silencing LINC00094 combined with MEM provides a novel target for the therapy of AD.

## 1 INTRODUCTION

Alzheimer's disease (AD) is a neurodegenerative disease characterized with progressive cognitive decline and behavioral impairment, which is the most common form of dementia. Some major pathologic features of AD have been identified, including the deposition of cerebrovascular amyloid‐beta (Abeta),^1^ the dysfunction of endothelial cell,^2^ the degeneration of cerebrovascular and neurons^3^ and the disruption of blood‐brain barrier (BBB).^4^, ^5^ The dysfunction of BBB may lead to impaired Abeta clearance and then an abnormal accumulation of Abeta plaques in various brain tissues, as well as the formation of pathological blood vessels. These results suggest that BBB dysfunction may eventually contribute to accelerate the progress of AD.^6^, ^7^

Under physiological conditions, the BBB is a dynamic interface that physically shields the exchanges of various substances between blood and the central nervous system (CNS). The BBB plays a pivotal role in the maintenance of CNS homeostasis and providing protection of brain against many toxic compounds.^8^ BBB consists of brain microvascular endothelial cells, basement membranes, and other surrounding cells in neurovascular unit such as pericytes, and perivascular astrocyte end‐feet.^8^ Moreover, tight junctions (TJs) between brain microvascular endothelial cells play critical roles in maintaining the integrity and permeability of the BBB.^9^

Memantine (MEM) is a low affinity, uncompetitive glutamatergic *N*‐methyl‐d‐aspartate (NMDA) receptor antagonist that has been widely used as a clinical practice for AD therapy.^10^ Growing evidence has shown that MEM could reduce the level of Abeta peptide in the cerebral cortex and culture endothelial cells of APP/PS1 transgenic mice.^11^ MEM also could contribute to the recovery of action potential in myelinated axons in pathological conditions.^12^ Another study highlights that MEM could rescue early SAH‐induced neurological impairment by improving impaired the permeability of BBB, inhibiting nNOS activity and peroxynitrite formation and subsequently suppressing apoptotic cascade.^13^ However, the mechanism of MEM on BBB permeability in AD microenvironment remains unclear.

Growing evidence is pointing towards that long non‐coding RNAs (lncRNAs) appear to regulate multiple biological processes and expression of these non‐coding molecules seems to be strictly regulated in physiological conditions as well as in several human disease.^14^ Current studies have shown that lncRNAs play an important role in neurodegenerative disease. LINC00094 is located on Chr9q34.2. It has been reported that LINC00094 may as a prognostic biomarker of lung cancer.^15^ Moreover, microarray analysis showed that LINC00094 is down‐regulated in MEM‐incubated cells. However, the regulatory role and potential mechanisms of LINC00094 affecting the BBB permeability have not been investigated.

Numerous miRNAs have been implicated in a variety of cellular progresses including cell proliferation, differentiation, apoptosis, stress response and metabolism,^16^ and their role in neurodegeneration has been widely reported.^17^, ^18^ It has been reported that lncRNAs may exert their function by sponging miRNAs. When LINC00094 was knocked down in ECs, microarray results showed that miR‐224‐5p and miR‐497‐5p were two of the up‐regulated miRNAs. Also, the bioinformatics database Starbase shows that LINC00094 harbors putative binding sites of miR‐224‐5p/miR‐497‐5p. It has been shown that miR‐224‐5p plays a critical role in multiple biological processes.^19^ Precious study has shown that miR‐224‐5p acted as a protective role in inner ear damage via targeting Ptx3.^20^ MiR‐497‐5p is one of the members of the miR‐15/107 family,^21^, ^22^ and the expression profile of miR‐497‐5p in vascular diseases has been extensive studied. Multiple studies shown that miR‐497‐5p is overexpressed and participates in regulating ECs function and neuroprotection.^23^ Yet, whether the cross‐regulation between LINC00094 and miR‐224‐5p/miR‐497‐5p affects BBB permeability in AD microenvironment remains unclear.

In our previous study, we demonstrated that Endophilin‐1 was up‐regulated in Abeta‐ECs. Remarkably, by searching bioinformatics databases Targetscan, we observed that Endophilin‐1 has putative binding sites for miR‐224‐5p/miR‐497‐5p. Endophilin‐1 (SH3GL2) belongs to the endocytosis protein family with a C‐terminal Src homology 3 (SH3) domains,^24^ which mainly expresses in adult frontal cortex and fetal cerebellum at 20 weeks gestation.^25^ Earlier studies have shown that endophlin‐1 played a crucial role in the regulation of the kidney glomerular filtration barrier via its endocytosis function.^26^ Furthermore, we previously demonstrated that Endophilin‐1 influenced the permeability of BBB by modulating the TJs‐related protein expression levels and redistribution of TJs‐related proteins ZO‐1 and occludin through the EGFR‐ERK1/2 and the EGFR‐JNK pathway.^27^, ^28^

In our present study, we first investigated the expression of LINC00094, miR‐224‐5p, miR‐497‐5p and Endophilin‐1 in ECs after Abeta_1‐42_ incubation. Then we clarified the changes of the above factors after MEM treatment. Furthermore, we confirmed the role of the above factors in BBB permeability and BBB integrity. We aim at providing a new target for AD treatment regard of BBB.

## 2 MATERIALS AND METHODS

### 2.1 Cell cultures

The human cerebral microvascular endothelial cell line hCMEC/D3 was a gift from Dr. Couraud (Institut Cochin, Paris, France). ECs were limited from 28 to 32 passages. Human brain vascular pericytes (HBVP) and normal human astrocytes (NHA) were obtained from the Sciencell Research Laboratories (Carlsbad, CA, USA). NHA and HBVP applied in this study were limited with passage below 10 and 12, respectively. The cells were cultured as described previously.^29^ Human embryonic kidney 293 (HEK293T) cells were acquired from Shanghai Institutes for Biological Sciences Cell Resource Center and the cell culture has been previously detailed.^30^ All cells were cultured in a humidified incubator at 37°C with 5% CO_2_.

Abeta_1‐42_ was obtained from Sigma‐Aldrich (St. Louis, MO, USA). Abeta_1‐42_ should be initially dissolved at a concentration of 2 mmol/L in dry DMSO and stored at −20°C. For oliomeric conditions, 2 mmol/L Abeta_1‐42_ in DMSO was diluted into 200 μmol/L in cold Opti‐MEM media and incubated at 4°C for 24 hours. MEM was gifted from H.Lundbeck A/S (Copenhagen‐Valby, Denmark) and dissolved in PBS. Cells were pre‐incubated with Abeta_1‐42_, MEM, or PBS as a control for 48 hours. The concentrations used in this work were 5 and 10 μmol/L, respectively.

### 2.2 In vitro BBB model establishment

The in vitro co‐culturing BBB model was established following Liu et al^29^ First, pericytes were seeded (2 × 10^5^ cells/cm^2^) onto the lower chamber of Transwell inserts (0.4 μm pore size; Corning, NY, USA). After pericytes cells were cultured overnight, 2 × 10^5^ cells/cm^2^ hCMEC/D3 cells were subsequent placed on the upper chambers of Transwell inserts. NHA (2 × 10^5^ cells/cm^2^) were seeded onto the 6‐well culture plate and cultured for 48 hours before adding ECs inserts.

### 2.3 Real‐time PCR assay

Trizol reagent (Thermo Fisher Scientific, Carlsbad, CA, USA) was applied to extract total RNA from cells. The expression levels of LINC00094 and GAPDH were detected by One‐Step SYBR PrimeScript RT‐PCR Kit (Perfect Real Time; Takara Bio, Inc., Kusatsu, Japan). The expression levels of miR‐224‐5p/miR‐497‐5p and U6 were detected by TaqMan miRNA Reverse Transcription kit and Taqman Universal Master Mix II (Applied Biosystems, Foster City, CA, USA). High Capacity cDNA Reverse Transcription Kits (Applied Biosystems) and TaqMan Universal Master Mix II were used to perform Endophilin‐1 and GAPDH expression. The relative quantification (2^−△△Ct^) method was performed to normalize and calculate relative gene expression values. Primers and probes were listed in Table S1.

### 2.4 Human lncRNA and miRNAs microarrays

LncRNA and miRNAs analysis, sample preparation, and microarray hybridization were performed by Kangchen Bio‐tech (Shanghai, China).

### 2.5 Cell transfection

Silencing plasmid of LINC00094 (NR_149319.1) was ligated into the pGPU6/GFP/Neo vector (GenePharma, Shanghai, China) to construct the shLINC00094 plasmid, and its non‐targeting sequences were used as a NC. The human Endophilin‐1 (Gene ID: 6456) gene coding sequence was ligated into pIRES2 vector (GenScript, Piscataway, NJ, USA) to construct the Endophilin‐1 overexpression plasmid, respectively. The respective no‐targeting sequences were used as NCs.

The ECs were seeded in 24‐well plates when the confluence reached at 50%‐80% and stable transfected via LTX and Plus reagent (Life Technologies, Carlsbad, CA, USA). Geneticin (G418; Sigma‐Aldrich) was performed to select the stable transfected cells. Then G418‐resistant cell clones were obtained after 4 weeks.

Furthermore, agomir‐224‐5p/agomir‐497‐5p (miR‐224‐5p (+)/miR‐497‐5p (+)), antagomir‐224‐5p/antagomir‐497‐5p (miR‐224‐5p (−)/miR‐497‐5p (−)) and their NC sequence (miR‐224‐5p (+) NC/miR‐497‐5p (+) NC and miR‐224‐5p (−) NC/miR‐497‐5p (−) NC; GenePharma) were transiently transfected into Abeta1‐42‐incubated ECs, which stably transfected shLINC00094 or Endophilin‐1 over‐expression with lipofectamine 3000 reagent, respectively. After 48 hours, the transiently transfected cells were obtained.

Sequences of shLINC00094 and shNC were shown in Table S2. The transfected efficiency of LINC00094, miR‐224‐5p (miR‐497‐5p) and Endophilin‐1 were shown in Figures S2 and S3.

### 2.6 Transendothelial electric resistance (TEER) assays

After establishing in vitro BBB models, TEER assay was performed by using a millicell‐ERS apparatus (Millipore, Billerica, MA, USA). Each measurement was placed in room temperature for 30 minutes before TEER assay was recorded. TEER assay was measured after the medium exchange. The final resistance (Ω·cm^2^) was calculated by subtracting background electrica resistance, and then multiplying by the effective surface area of the transwell insert.

### 2.7 Horseradish peroxidase (HRP) flux measurement

The permeability of the in vitro BBB models was detected by Horseradish Peroxidate (HRP) flux. After the BBB models were constructed, 1ml of serum‐free EBM‐2 medium containing 10 μg/mL HRP (0.5 mmol/L, Sigma‐Aldrich) culture medium was added into the upper champer of the transwell system. One hour later, 5 μL of culture medium in the lower chamber was collected and the HRP content of the samples was detected by TMB colorimetry approach. The final HRP flux was expressed as pmol/cm^2^/h.

### 2.8 Western blot assays

Equal amounts of proteins were further separated using SDS‐PAGE and then transferred to polyvinylidene fluoride (PVDF) membranes (Millipore). Membranes were blocked to avoid non‐specific bindings in Tris‐buffered saline‐Tween (TBST) containing 5% fat‐free milk for 2 hours. Subsequently incubated with primary antibodies (Endophilin‐1, 1:250, Santa Cruz Biotechnology, Dallas, Texas, USA, sc‐374278; ZO‐1, 1:600, Life Technologies Corp, 61‐3700; occludin, 1:600, Abcam, Cambridge, UK, ab31721; claudin‐5, 1:500, Thermo Fisher Scientific, 34‐1600; β‐actin, 1:2000, Abcam, ab8226) at 4 °C overnight. After three washes, membranes were incubated with the corresponding secondary antibody at a 1:10 000 dilution at room temperature for 2 hours. After washing, immunoblots were visualized by enhanced chemiluminescence (ECL kit, Santa Cruz Biotechnology). All the protein bands were scanned by Chem Imager 5500 V2.03 software and the integrated density values (IDVs) were calculated utilizing FluorChem 2.0 software.

### 2.9 Immunofluorescence assays

Cells on glass coverslips were fixed by 4% paraformaldehyde for 20 minutes, and blocked via 5% bovine serum album in phosphate‐buffered saline for 2 hours at room temperature. Cells were incubated with primary antibodies (1:50 anti‐ZO‐1; 1:50 anti‐occludin; 1:50 anti‐claudin‐5) respectively at 4°C overnight. After three washes with phosphate‐buffered saline, cells were incubated for 2 hours with fluorophore‐conjugated secondary antibodies. DAPI was applied to visualize cell nuclei. The staining was analyzed via Olympus DP71 immunofluorescence microscope (Olympus, Tokyo, Japan) and merged with Chemi Imager 5500 V2.03 software.

### 2.10 Reporter vector construction and Luciferase reporter assay

The putative target binding sequences of miR‐224‐5p/miR‐497‐5p in LINC00094 and Endophilin‐1 3′‐UTR gene and their mutant sequences were synthesized and cloned into the pmirGLO dual‐luciferase vector (Promega, Madison, WI, USA). Wild‐type and mutated LINC00094 or Endophilin‐1‐3′UTR reporter plasmid and agomir‐224‐5p/agomir‐497‐5p or agomir‐224‐5p‐NC/agomir‐497‐5p‐NC were co‐transfected into HEK293T cells. The pmirGLO empty vector was transfected as a Control. The luciferase activity was measured 48 hours after transfection by the Dual‐Luciferase Reporter System (Promega).

### 2.11 RNA immunoprecipitation (RIP) assay

Magna RNA‐binding protein immunoprecipitation kit (Millipore) was used to perform RIP assay. Whole cell lysate was incubated with human anti‐Ago2 antibody, or NC normal mouse IgG. Furthermore, purified RNA was extracted and applied to qRT‐PCR to demonatrate the presence of the binding targets.

### 2.12 Satistical analysis

Statistical analysis was performed with GraphPad Prism v5.01 (GraphPad Software, La Jolla, CA, USA) software. Data was described as mean ± standard deviation (SD). Student's *t* test was used for comparisons between two groups. One‐way ANOVA was used for multi‐group comparisons followed by Bonferroni's post‐test. Difference was considered to be statistically significant if *P < *0.05.

## 3 RESULTS

### 3.1 MEM decreased the expression of LINC00094 in Abeta_1‐42_‐incubated ECs and decreased BBB permeability in AD microenvironment

**Figure 1:**
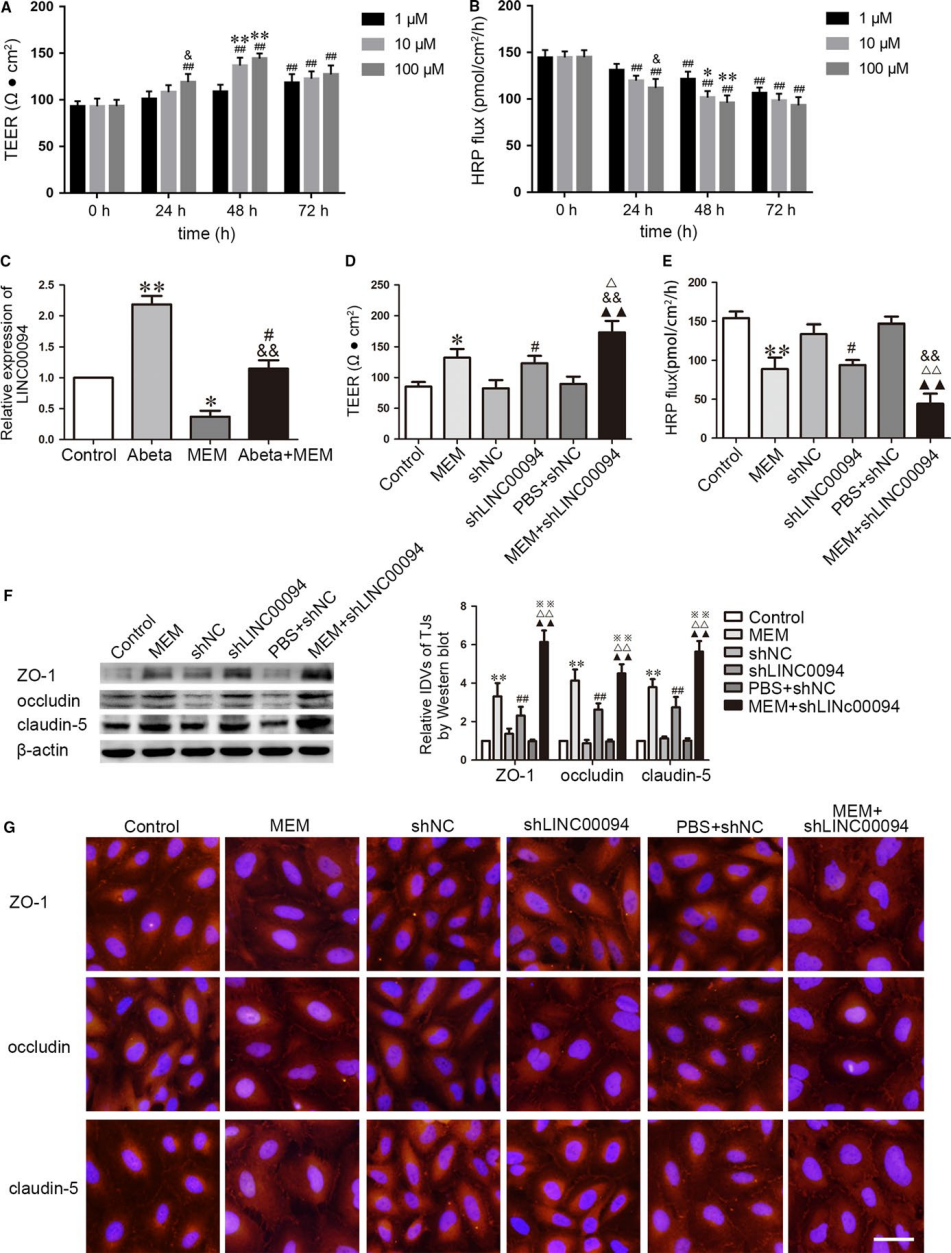
MEM decreased the expression of LINC00094 in Abeta_1‐42_‐incubated ECs, increased the expression of ZO‐1, occludin and claudin‐5, and decreased BBB permeability in AD microenvironment. (A, B) The effects of MEM on TEER values and HRP flux of Abeta_1‐42_‐incubated ECs after treatment with 1, 10 and 100 μmol/L for 24, 48 and 72 hours. Data represent mean ± SD (n = 3, each). ^##^ *P *< 0.01 vs 0 hour group, *P < 0.05, **P < 0.01 vs 1 μmol/L 48 hours, ^&^ *P *< 0.05 vs 1 μmol/L 24 hours. (C) Relative mRNA expression of LINC00094 in ECs pre‐incubated with Abeta_1‐42_, MEM and Abeta_1‐42_+MEM by qRT‐PCR. Data represent mean ± SD (n = 3, each). *P < 0.05, **P < 0.01 vs Control group, ^&&^ *P *< 0.01 vs Abeta_1‐42_ group, ^#^ *P *> 0.05 vs Control group. (D) TEER values of Abeta_1‐42_‐incubated ECs were detected to confirm BBB integrity. (E) HRP flux test was performed to confirm BBB permeability. (F) Western blot analysis to determine the expression of ZO‐1, occludin and claudin‐5 in Abeta_1‐42_‐incubated ECs. Data represent mean ±SD (n = 3, each). *P < 0.05, **P < 0.01 vs Control group, ^##^ *P *< 0.01 vs shNC group, ^△^ *P *< 0.05, ^△△^ *P *< 0.01 vs MEM group, ^&&^ *P *< 0.01 vs shLINC00094 group, ^▲▲^ *P *< 0.01 vs PBS + shNC group. (G) Immunofluorescence assay was used to analyze the expression and distribution of ZO‐1, occludin and claudin‐5. Images were representative of three independent experiments. Scale bar represents 30 μm

To test the effects of MEM on BBB permeability in AD microenvironment, we firstly evaluated TEER values and HRP flux at indicated time and concentration. As shown in Figure 1A,B, MEM regulated BBB permeability in a dose‐ and time‐dependent manner. Compared with 0 hours group, TEER values were significantly increased and HRP flux was decreased at 48 hours with the treatment of 10 and 100 μmol/L MEM (*P *< 0.01), whereas there was no obvious difference among these two groups (*P *> 0.05). Thus 10 μmol/L at 48 hours were selected as the optimum concentration and time point in the subsequent experiments. We further performed microarray analysis to assess the expression patterns of lncRNAs in MEM‐incubated ECs. As shown in Figure S1A, LINC00094, LINC00052, LINC00312 and LINC00625 are four of the most abundant lncRNAs in MEM‐incubated ECs. Meanwhile, qRT‐PCR analysis showed that expression levels of LINC00094, LINC00052, LINC00312 and LINC00625 were significantly declined in MEM‐incubated ECs (Figure S1C). Subsequently, we tested all the candidates’ transfected efficiency of knockdown and performed loss‐of‐function tests to clarify the potential roles of these lncRNAs on BBB permeability in AD microenvironment. Remarkably, downregulation of LINC00094 increased TEER values and decreased HRP flux in BBB models in vitro. However, LINC00052, LINC00312, LINC00625 showed few effects on TEER values and HRP flux (Figure S2). Accordingly, LINC00094 was selected to perform the subsequent analyses.

As shown in Figure 1C, “Control” group means normal endothelial cells, “Abeta” group means Abeta‐incubated ECs, “MEM” group means MEM‐incubated ECs and “Abeta + MEM” group means ECs co‐incubated with Abeta_1‐42_ + MEM. LINC00094 was significantly up‐regulated in Abeta_1‐42_‐incubated ECs (*P *< 0.01) and down‐regulated in MEM‐incubated ECs (*P *< 0.01). The expression of LINC00094 in ECs co‐incubated with Abeta_1‐42_ + MEM largely reversed the Abeta_1‐42_‐incubated induced increase of LINC00094 expression. We further investigate LINC00094 function by stable transfection of shLINC00094 and establish BBB model in vitro. As shown in Figure 1D‐G, “Control” group means Abeta‐incubated ECs. TEER value was higher in the MEM‐incubated ECs group than Control group, suggesting that MEM repaired BBB integrity (*P *< 0.05, Figure 1D). The penetration rate of HRP was lower in the MEM‐incubated ECs group compared with the Control group (Figure 1E), which indicated that MEM decreased BBB permeability. Compared with shNC group, the shLINC00094 group exhibited an increase in TEER value (*P *< 0.01, Figure 1D), and a decrease in HRP flux (*P *< 0.01, Figure 1E). The TEER value in MEM + shLINC00094 group was higher than that in MEM, shLINC00094 and PBS + shNC group. The HRP flux was lower than that in MEM, ShLINC00094 and PBS + shNC group. The expression of TJ‐related proteins ZO‐1, occludin and claudin5 in ECs was detected by Western blot assay. Results showed that the expression of TJs in the MEM‐incubated combined with silencing LINC00094 group presented higher expression compared with that in the Control group and shNC group respectively (Figure 1F). Similarly, immunofluorescence assays revealed that MEM, LINC00094 inhibition promoted the expression of TJs, with the continuous liner distribution on the boundaries of ECs, while the change of MEM + shLINC00094 group was the most obvious (Figure 1G). These results revealed that MEM decreased the permeability of BBB in AD microenvironment by knockdown of LINC00094.

### 3.2 LINC00094 influenced BBB permeability via binding to miR‐224‐5p/miR‐497‐5p

**Figure 2:**
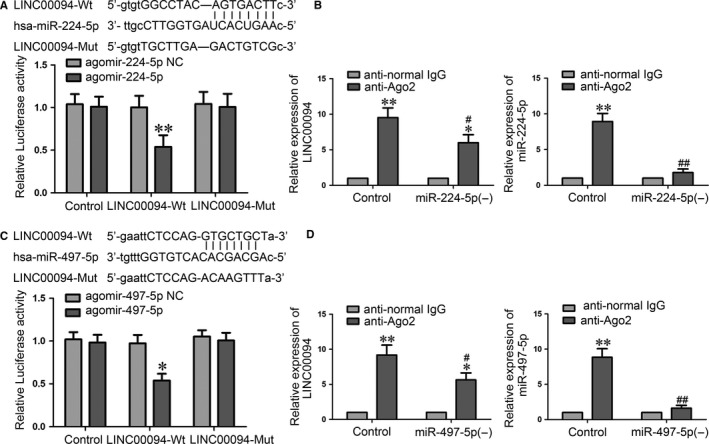
MiR‐224‐5p/miR‐497‐5p were sponged by LINC00094. (A, C) Dual‐luciferase reported assay was used to perform the relative luciferase activity. Data represent mean ± SD (n = 3, each). **P < 0.01 vs LINC00094‐Wt + agomir‐224‐5p NC group, *P < 0.05 vs LINC00094‐Wt + agomir‐497‐5p NC group. (B, D) RIP assay were performed with normal mouse IgG or anti‐Ago2. Relative expression levels of LINC00094 and miR‐224‐5p/miR‐497‐5p were determined by qRT‐PCR. Data represent mean ± SD (n = 3, each). *P < 0.05, **P < 0.01 vs anti‐normal IgG respective group, ^#^ *P *< 0.05, ^##^ *P *< 0.01 vs anti‐Ago2 in Control group

LncRNA may modulate the biological function of miRNAs as a competing endogenous RNA or miRNA “sponge”.^31^, ^32^ Using miRNAs microarrays, we found miR‐224‐5p/miR‐497‐5p was significantly upregulated in ECs treated with shLINC00094 (Figure S1B,D). To confirm whether miR‐224‐5p/miR‐497‐5p is regulated by LINC00094, we further performed a dual‐luciferase reporter assay and RIP assay. The dual‐luciferase reporter assay indicated that the co‐transfection of pmirGLO‐LINC00094‐Wt and agomir‐224‐5p (agomir‐497‐5p) results in lower luciferase activity compared with the co‐transfection of pmirGLO‐LINC00094‐Mut and agomir‐224‐5p (agomir‐497‐5p) (Figure 2A,C). The RIP assay showed that LINC00094 and miR‐224‐5p/miR‐497‐5p were higher in anti‐Ago2 immunoprecipitates compared with anti‐IgG group. The down‐regulation of miR‐224‐5p (miR‐497‐5p) decreased the expression of LINC00094 and miR‐224‐5p (miR‐497‐5p) immunoprecipitated with Ago2 (Figure 2B,D). These data indicate that LINC00094 regulated miR‐224‐5p/miR‐497‐5p function by ‘sponging’ it.

**Figure 3:**
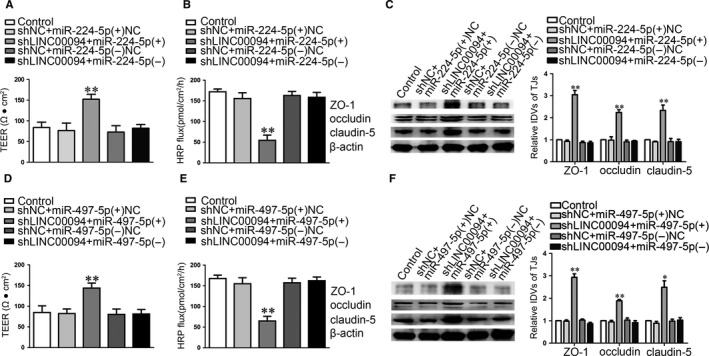
MiR‐224‐5p/miR‐497‐5p were involved in LINC00094 mediated the effects on Abeta_1‐42_‐incubated ECs. (A, D) TEER values of Abeta_1‐42_‐incubated ECs were detected to confirm BBB integrity. (B, E) HRP flux test was performed to confirm BBB permeability. (C, F) Western blot analysis was used to determine the expression of ZO‐1, occludin and claudin‐5 in Abeta_1‐42_‐incubated ECs. Data represent mean ± SD (n = 3, each). *P < 0.05，**P < 0.01 vs shNC + miR‐224‐5p (+) NC/shNC + miR‐497‐5p (+) NC group

To investigate the role of miR‐224‐5p/miR‐497‐5p on LINC00094‐regulated BBB permeability, stable LINC00094 silencing ECs were transfected with miR‐224‐5p/miR‐497‐5p agomir or antagomir. AS shown in Figure 3, “Control” group means Abeta‐incubated ECs. The results showed that the down‐regulation of miR‐224‐5p (miR‐497‐5p) markedly reversed the LINC00094 knockdown induced increase in TEER and decrease in HRP flux (Figure 3A,B,D,E). Similarly, the Western blot assays revealed that the higher expression of TJs (Figure 3C,F) induced by scienced‐LINC00094 was reversed by the shLINC00094 + miR‐224‐5p (−)/shLINC00094 + miR‐497‐5p (−) group.

### 3.3 MiR‐224‐5p/miR‐497‐5p overexpression increased the expression of TJ‐related proteins, meanwhile decreased BBB permeability in AD microenvironment

**Figure 4:**
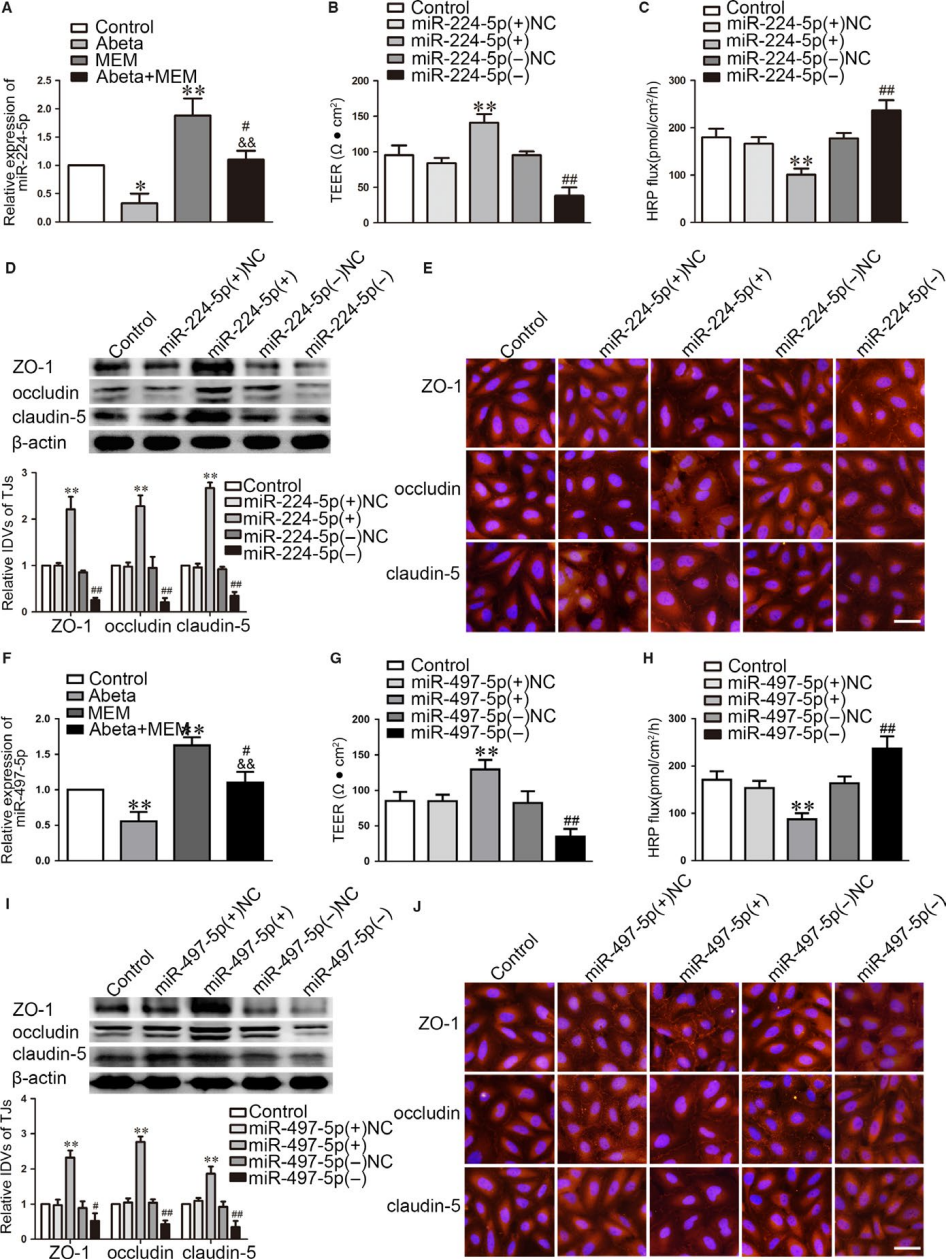
MiR‐224‐5p/miR‐497‐5p over‐expression increased the expression of ZO‐1, occludin and claudin‐5, and decreased BBB permeability in AD microenvironment. (A, F) Relative mRNA expressions of miR‐224‐5p (A) and miR‐497‐5p (F) in ECs pre‐incubated with Abeta_1‐42_, MEM and Abeta_1‐42_ + MEM were detected by qRT‐PCR. Data represent mean ± SD (n = 3, each). *P < 0.05, **P < 0.01 vs Control group, ^&&^ *P *< 0.01 vs Abeta_1‐42_ group, ^#^ *P *> 0.05 vs Control group. (B, G) TEER values of Abeta_1‐42_‐incubated ECs were detected to confirm BBB integrity. (C, H) HRP flux test was performed to confirm BBB permeability. (D, I) Western blot analysis was used to determine the expression of ZO‐1, occludin and claudin‐5 in Abeta_1‐42_‐incubated ECs. Data represent mean ± SD (n = 3, each). **P < 0.01 vs miR‐224‐5p/miR‐497‐5p (+) NC group, ^##^ *P *< 0.01 vs miR‐224‐5p/miR‐497‐5p (−) NC group. (E, J) Immunofluorescence assay was used to analyze the expression and distribution of ZO‐1, occludin and claudin‐5. Images were representative of three independent experiments. Scale bar represents 30 μm

To further clarify the role of miR‐224‐5p/miR‐497‐5p in BBB permeability in AD microenvironment, we first detected the expression of miR‐224‐5p/miR‐497‐5p in Abeta_1‐42_‐incubated ECs. As shown in Figure4A,F, “Control” group means normal endothelial cells, “Abeta” group means Abeta‐incubated ECs, “MEM” group means MEM‐incubated ECs and “Abeta+MEM” group means ECs co‐incubated with Abeta_1‐42_ + MEM. The results as shown in Figure 4A,F, miR‐224‐5p/miR‐497‐5p were down‐regulated in Abeta_1‐42_‐incubated ECs and up‐regulated in MEM‐incubated ECs. The combination of the two combinations largely reversed Abeta_1‐42_‐induced miR‐224‐5p/miR‐497‐5p decreased (*P *< 0.01). AS shown in Figure 4B‐E, G‐J, “Control” group means Abeta‐incubated ECs. Further, TEER and HRP flux test were used to determine the functional roles of miR‐224‐5p (miR‐497‐5p) on the integrity and permeability of BBB. MiR‐224‐5p (+)/miR‐497‐5p (+) group exhibited an increase in TEER and a decrease in HRP flux. MiR‐224‐5p (−)/miR‐497‐5p (−) group exhibited opposite effects (Figure 4B,C,G,H).

The mechanistic studies shown that the expression levels of TJs were up‐regulated in the miR‐224‐5p (+)/miR‐497‐5p (+) group compared with miR‐224‐5p (+) NC/miR‐497‐5p (+) NC group respectively, whereas miR‐224‐5p (−)/miR‐497‐5p (−) produced the opposite results (Figure 4D,I). Subsequent immunofluorescence demonstrated that TJs showed a continuous distribution along the cell‐cell boundaries, and were abundant in expression in miR‐224‐5p (+)/miR‐497‐5p (+) group. Opposite effects were observed in the miR‐224‐5p (−)/miR‐497‐5p (−) group (Figure 4E,J).

### 3.4 MiR‐224‐5p/miR‐497‐5p inhibited the expression of Endophilin‐1 by binding to Endophilin‐1 mRNA 3′‐UTR

**Figure 5:**
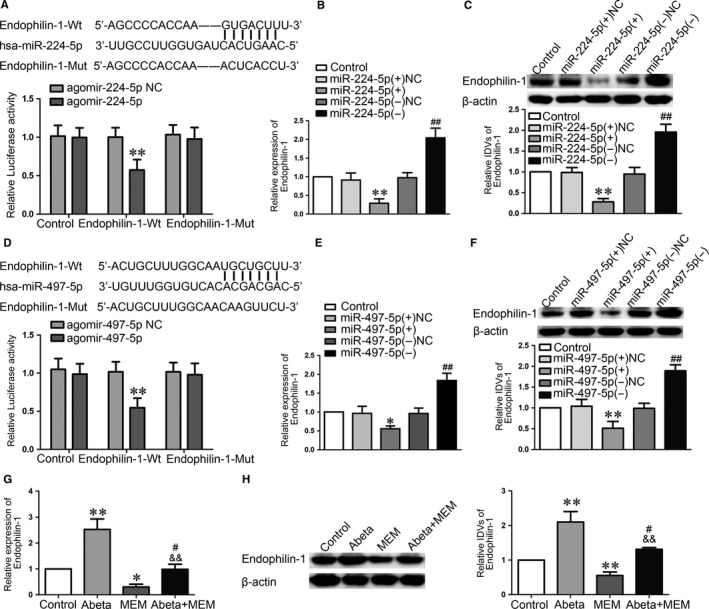
MiR‐224‐5p/miR‐497‐5p targeted Endophilin‐1. (A, D) Dual‐luciferase reported assay was used to perform the relative luciferase activity. Data represent mean ± SD (n = 3, each). **P < 0.01 vs Endopilin‐1‐Wt + agomir‐224‐5p NC/Endopilin‐1‐Wt + agomir‐497‐5p NC group. (B, E) qRT‐PCR was used to detect the relative mRNA expression level of Endophilin‐1 in Abeta_1‐42_‐incubated ECs. (C, F) Western blot analysis was used to determine the expression of Endophilin‐1 in Abeta_1‐42_‐incubated ECs. Data represent mean ± SD (n = 3, each). *P < 0.05，**P < 0.01 vs miR‐224‐5p (+) NC/miR‐497‐5p (+) NC group, ^##^ *P *< 0.01 vs miR‐224‐5p (−) NC/miR‐497‐5p (−) NC group. (G) qRT‐PCR was used to detect the relative mRNA expression level of Endophilin‐1 in ECs with Abeta_1‐42_, MEM and Abeta_1‐42_ + MEM by qRT‐PCR. Data represent mean ± SD (n = 3, each). *P < 0.05，**P < 0.01 vs Control group, ^&&^ *P *< 0.01 vs Abeta_1‐42_ group, ^#^ *P *> 0.05 vs Control group. (H) Western blot analysis was used to determine the expression of Endophilin‐1 in ECs pre‐incubated with Abeta_1‐42_, MEM and Abeta_1‐42_ + MEM. Data represent mean ± SD (n = 3, each). **P < 0.01 vs Control group, ^&&^ *P *< 0.01 vs Abeta_1‐42_ group, ^#^ *P *> 0.05 vs Control group

We found that miR‐224‐5p/miR‐497‐5p have putative binding sites with the Endophilin‐1 3′‐UTR respectively, which indicated that miR‐224‐5p/miR‐497‐5p may directly regulates Endophilin‐1. Subsequent dual‐luciferase reporter assays showed that the Endophilin‐1‐Wt + agomir‐224‐5p/agomir‐497‐5p impaired luciferase activity compared with Endophilin‐1‐Mut + agomir‐224‐5p/agomir‐497‐5p (Figure 5A,D). We further explored the expression of Endophilin‐1. AS shown in Figure 5B,C,E,F, “Control” group means Abeta‐incubated ECs. Results revealed that the mRNA and protein expression of Endophilin‐1 were inhibited in the miR‐224‐5p (+)/miR‐497‐5p (+) group (Figure 5B,C,E,F).

### 3.5 MiR‐224‐5p/miR‐497‐5p over‐expression increased the expression of ZO‐1, occludin and claudin‐5, and decreased BBB permeability in AD microenvironment via regulating Endophilin‐1 function

**Figure 6:**
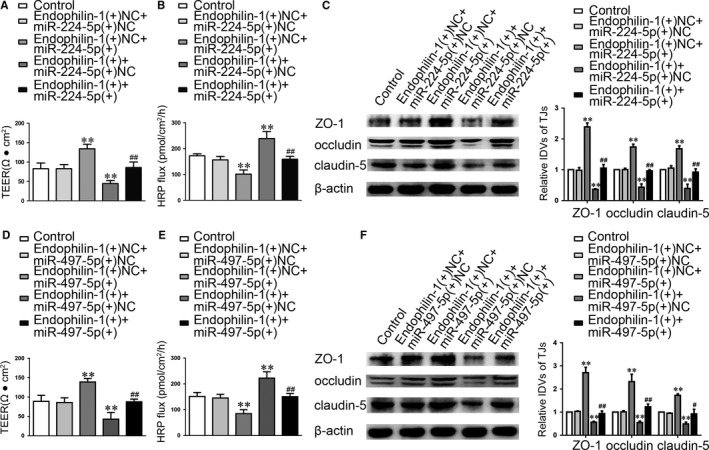
Endophilin‐1 mediated the effect of miR‐224‐5p/miR‐497‐5p over‐expression on ECs. (A, D) TEER values of Abeta_1‐42_‐incubated ECs were detected to confirm BBB integrity. (B, E) HRP flux test was performed to confirm BBB permeability. (C, F) Western blot analysis was used to determine the expression of ZO‐1, occludin and claudin‐5 in Abeta_1‐42_‐incubated ECs. Data represent mean ± SD (n = 3, each). **P < 0.01 vs Endophilin‐1 (+) NC + miR‐224‐5p (+) NC/Endophilin‐1 (+) NC + miR‐497‐5p (+) NC group, ^#^ *P* < 0.05，^##^ *P* < 0.01 vs Endophilin‐1 (+) NC + miR‐224‐5p (+)/Endophilin‐1 (+) NC + miR‐497‐5p (+) group

We next explored whether Endophilin‐1 was involved in MEM‐mediated regulation of BBB permeability in AD microenvironment. As shown in Figure 5G,H, “Control” group means normal endothelial cells, “Abeta” group means Abeta‐incubated ECs, “MEM” group means MEM‐incubated ECs and “Abeta + MEM” group means ECs co‐incubated with Abeta_1‐42_ + MEM. The results shown that, Endophilin‐1 mRNA and protein expression were up‐regulated in Abeta_1‐42_‐incubated ECs, and those were down‐regulated in MEM‐incubated ECs. AS shown in Figure 6, “Control” group means Abeta‐incubated ECs. Compared with Abeta_1‐42_ group, the co‐incubated of Abeta_1‐42_ + MEM group significantly decreased the expression levels of Endophilin‐1. Co‐overexpression of Endophilin‐1 and miR‐224‐5p/miR‐497‐5p reversed the effect of miR‐224‐5p/miR‐497‐5p over‐expression alone on TEER and HRP flux (Figure 6A,B,D,E). Western blot assays showed that the co‐overexpression Endophilin‐1 and miR‐224‐5p/miR‐497‐5p largely reversed the TJs expression increased in ECs by miR‐224‐5p/miR‐497‐5p over‐expression alone (Figure 6C,F). These data indicate that Endophilin‐1 is involved in the miR‐224‐5p/miR‐497‐5p regulated BBB permeability.

**Figure 7:**
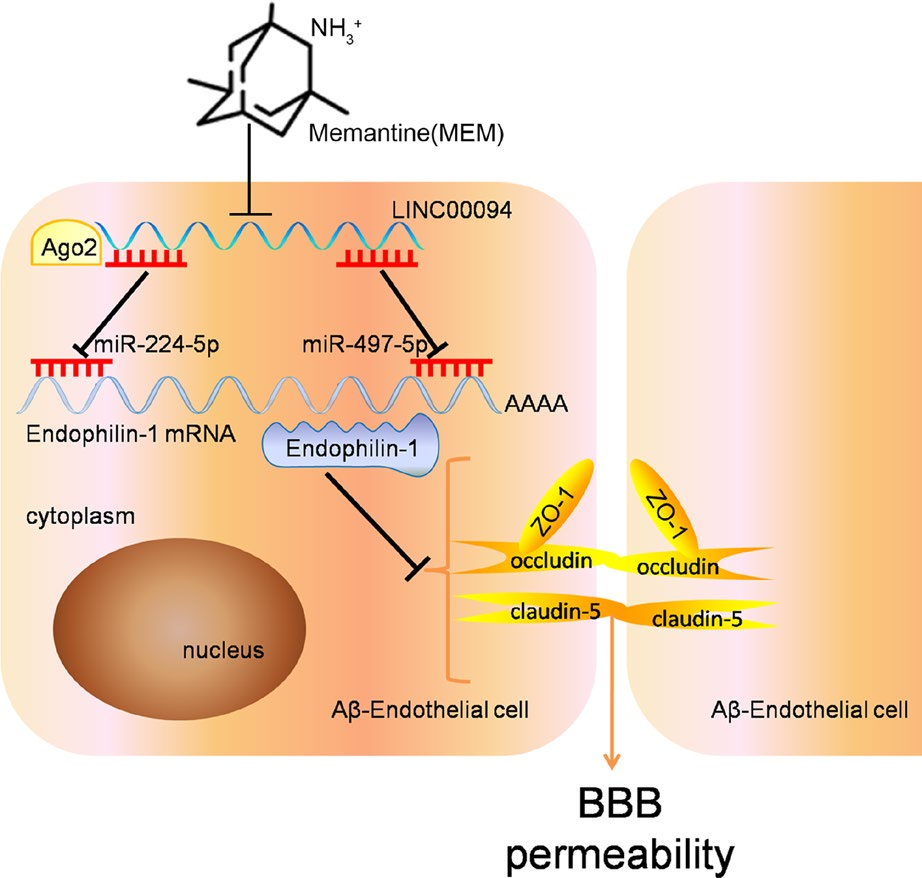
The schematic representation of MEM/LINC00094/miR‐224‐5p (miR‐497‐5p)/Endophilin‐1 axis in BBB permeability of AD microenvironment

## 4 DISCUSSION

In this study, we firstly demonstrated that MEM treatment contributed to ameliorate BBB permeability in AD microenvironment. Subsequently, LINC00094 was endogenously expressed in ECs of BBB model in vitro, while it was significantly up‐regulated in Abeta_1‐42_‐incubated ECs. Besides, it was down‐regulated in MEM‐incubated ECs of BBB models in vitro. MEM treatment and silencing LINC00094 decreased the permeability of BBB in AD microenvironment, with the effect of combined application being the most significant. Moreover, miR‐224‐5p/miR‐497‐5p was down‐regulated in Abeta_1‐42_‐incubated ECs, while they were up‐regulated in MEM‐incubated ECs. The analysis of the mechanism demonstrated that reduction of LINC00094 inhibited Endophilin‐1 expression by up‐regulating miR‐224‐4p/miR‐497‐5p, promoted the expression of TJs, and ultimately alleviated BBB permeability in AD microenvironment.

The BBB is a metabolic barrier that regulates materials exchange between the blood and CNS,^33^ which is of great significance to maintain brain homeostasis and its normal function.^34^, ^35^ TJs are comprised of the ZOs, occludin as well as claudins, which are considered the basic components responsible for proper function and integrity of BBB.^36^ Our research has displayed that MEM restored BBB permeability in AD microenvironment by up‐regulating the expression of TJ‐related proteins. Additionally, MEM reduces APP secretion in SK‐N‐SH human neuroblastoma cells and lowers the levels of Abeta peptides in APP/PS1 transgenic mice and cultured cortical cells via acting on γ‐secretase to improve spatial learning in APP/PS1 transgenic mice.^11^ A recent study investigated that a novel neuroprotective mechanism of MEM on neurodegenerative disease, that pretreatment with low‐dose MEM significantly prevents the attachment of monocyte to human brain microvascular endothelial cells (HBMECs) and ameliorates TNF‐α induced disruption of BBB in vitro model.^37^ However, little information regarding the roles of MEM on regulating BBB permeability in AD microenvironment has been reported to date. We demonstrated for the first time that MEM can reduce the permeability of BBB in AD microenvironment via increasing the expression of TJs. It provides a new experimental basis for the treatment of AD with MEM, suggesting that MEM might be involved in the regulation of BBB function in AD.

Accumulated evidence indicates that dysregulation or mutation of lncRNAs is tightly involved in diverse cellular process.^38^ It is urgent to ascertain the dysregulated lncRNAs and the underlying mechanism in a variety of neurodegenerative disorders. BC200 RNA was found to be up‐regulated in AD brain tissues, which regulating gene expression at translational level during the development of AD by interacting with many different proteins.^39^ We concerned that LINC00094 was up‐regulated in Abeta_1‐42_‐incubated ECs and down‐regulated in MEM‐induced ECs. Treatment with MEM and knockdown of LINC00094 alleviated the permeability of BBB selectively by up‐regulating TJs expressions in Abeta_1‐42_‐incubated ECs. In addition, combination of MEM and silencing LINC00094 significantly decreased the BBB permeability in AD microenvironment. However, the molecular mechanisms deserve further study.

Emerging evidence indicated that certain lncRNAs can served as a competitive endogenous RNA (“ceRNA”) to regulate downstream gene expression and biological function.^40^ For instance, RNCR3 acts as a ceRNA, and form a feedback loop with Kruppel‐like factor 2 and miR‐185‐5p to prevent atherosclerosis.^41^ TGFB2‐OT1 regulates autophagy in vascular endothelial cells (VECs) via sponging miR‐3960, miR‐4488, miR‐4459.^42^ Further, our search of miRanda revealed that miR‐224‐5p and miR‐497‐5p can bind to LINC00094 via the putative microRNA response elements (MREs). MRE has been identified to be a highly conserved sequence and used as a new language to explore ceRNA regulation network.^43^ The results of Dual‐luciferase reporter assay and RIP assay showed that miR‐224‐5p (miR‐497‐5p) was enriched by LINC00094 and sponge LINC0094 in a sequence‐specific manner, respectively. These results supported the hypothesis that LINC00094 regulates BBB permeability in AD microenvironment.via sponging miR‐224‐5p/miR‐497‐5p.

Multiple miRNAs are proved to be expressed abnormally in the CNS^44^, ^45^ and they have been implicated in a wide range of pathophysiological processes such as neurodegenerative disease.^46^, ^47^ For instance, miR‐34a is enriched in the cerebral cortex of AD mouse models and contributes to the pathogenesis of AD.^48^ Our previous study described that miR‐107 is down‐regulated in Abeta‐incubated ECs. Over‐expression of miR‐107 could alleviate BBB permeability and protect BBB integrity by up‐regulating TJs.^29^ In the present study, we demonstrated that miR‐224‐5p/miR‐497‐5p was highly expressed in Abeta_1‐42_‐incubated ECs and miR‐224‐5p/miR‐497‐5p over‐expression reduced BBB permeability in AD microenvironment by promoting TJs expression. Consistent with our results, miR‐155, miR‐181c, and miR‐29c regulate BBB function via targeting TJ‐related proteins or affecting related signal pathways.^49^ MiR‐Let7A significantly prevented loss of TJ‐related proteins and alleviates the cell apoptosis under high glucose in vitro condition, which contributed to ameliorate the disruption of BBB in hyperglycemia condition.^50^

In an attempt to elucidate the effect of miR‐224‐5p/miR‐497‐5p on BBB function, we further investigated its possible mechanism in regulating the integrity and the permeability of BBB in AD microenvironment. We employed bioinformatics tools and performed a dual‐luciferase assay. The results showed that Endophilin‐1 is a novel target of miR‐224‐5p (miR‐497‐5p). Endophilin‐1 plays a crucial role in the maintenance of the kidney glomerular filtration barrier.^26^ Additionally, Endophilin‐1 was increased in Abeta_1‐42_‐incubated ECs, which is consistent with our previous study.^29^ Besides, Endophilin‐1 was decreased in MEM‐incubated ECs. Co‐overexpression of Endophilin‐1 and miR‐224‐5p (miR‐497‐5p) reversed the effect of miR‐224‐5p (miR‐497‐5p) over‐expression alone in decreasing BBB permeability. Besides, Endophilin‐1 was identified as a key regulator in BBB and acted a key role in the protein expression levels of TJs.

In conclusion, we demonstrate for the first time that the expressions of LINC00094 and Endophilin‐1 were increased and miR‐224‐5p/miR‐497‐5p expressions were decreased in Abeta_1‐42_‐incubated ECs. In addition, MEM treatment might up‐regulate the expressions of ZO‐1, occludin and claudin‐5 via the LINC00094/miR‐224‐5p (miR‐497‐5p)/Endophilin‐1 axis, which contributed to ameliorate BBB permeability in AD microenvironment. Our present findings provide a new experimental basis for the treatment of AD on the perspective of BBB.

## CONFLICT OF INTEREST

The authors disclose that they have no competing interest.

## AUTHORS’ CONTRIBUTIONS

XS and YX conceived and designed the project. LZ, ML, JM performed most of the experiments. ML and WL performed statistics analysis. LG and SW helped with Immunofluorescence Assays. LZ and WL wrote the manuscript. XS and YX contributed to the manuscript revision. All authors had final approval of the submitted versions.

## AVAILABILITY OF DATA AND MATERIALS

The datasets during and/or analyzed during the current study are available from the corresponding author on reasonable request.
