# Supplementary material for: The role of LINC00094/miR‐224‐5p (miR‐497‐5p)/Endophilin‐1 axis in Memantine mediated protective effects on blood‐brain barrier in AD microenvironment
Source: J Cell Mol Med. 2019 Feb 22;23(5):3280–92. doi: 10.1111/jcmm.14214 (PMC6484416; doi:10.1111/jcmm.14214)
Supplement: Supplementary file 1 [file JCMM-23-3280-s001.pdf]

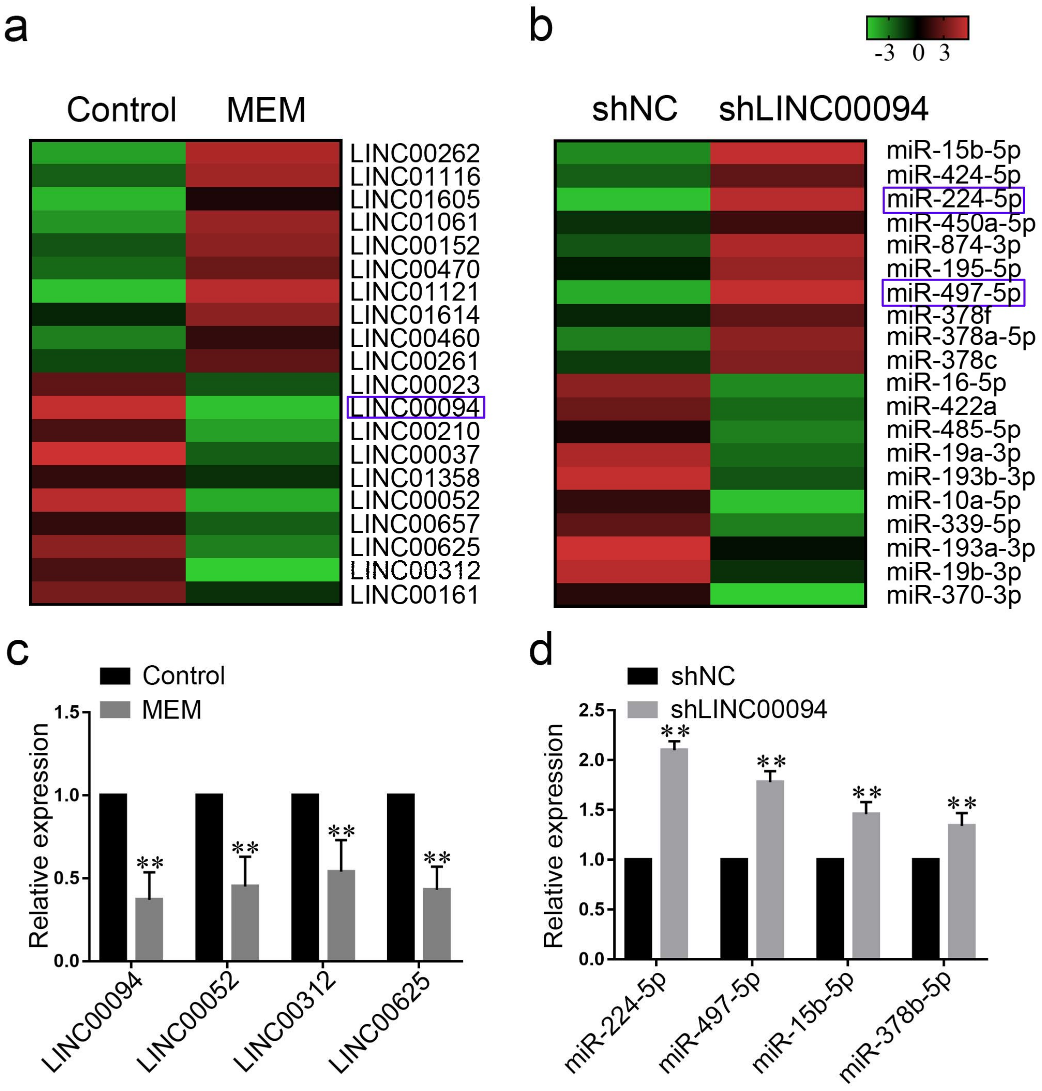

Figure S1. LncRNA and miRNA microarrays data in Control and MEM-incubated ECs. “Control” group means normal ECs and “MEM” group means MEM-incubated ECs. **(a)** LncRNA gene expression profiles as obtained from samples in three groups as indicated. **(b)** miRNA gene expression profiles as obtained from samples in three groups as indicated. **(c and d)** qRT-PCR was performed to validate the selected molecules. Data represent mean ± SD (n=3, each). $^{**}P < 0.01$ vs Control group/ shNC group.

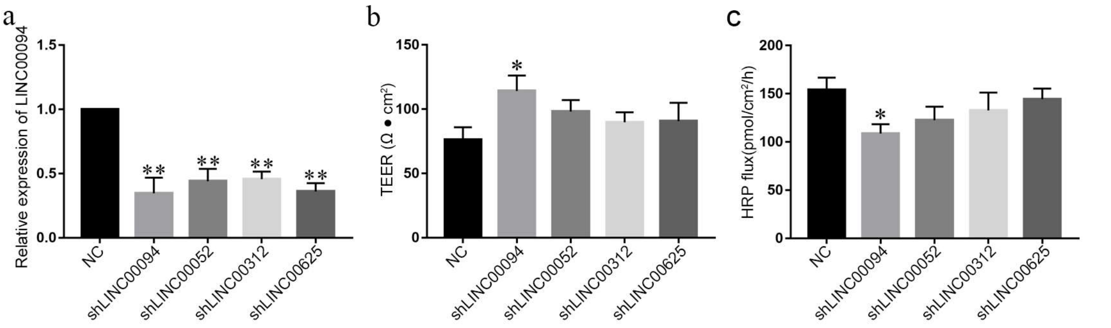

Figure S2. Effects of LINC00094, LINC00052, LINC00312 and LINC00625 knockdown on BBB permeability in AD microenvironment. **(a)** Relative expression of LINC00094, LINC00052, LINC00312 and LINC00625 in cells transfected shLINC00094, shLINC00052, shLINC00312 and shLINC00625. Data represent mean ± s.d. (n=3, each). **(b)** TEER values were detected to confirm BBB integrity. **(c)**HRP flux test was performed to confirm BBB permeability. Data represent mean ± SD (n=3, each). $^{*}P < 0.05$, $^{**}P < 0.01$ vs NC group.

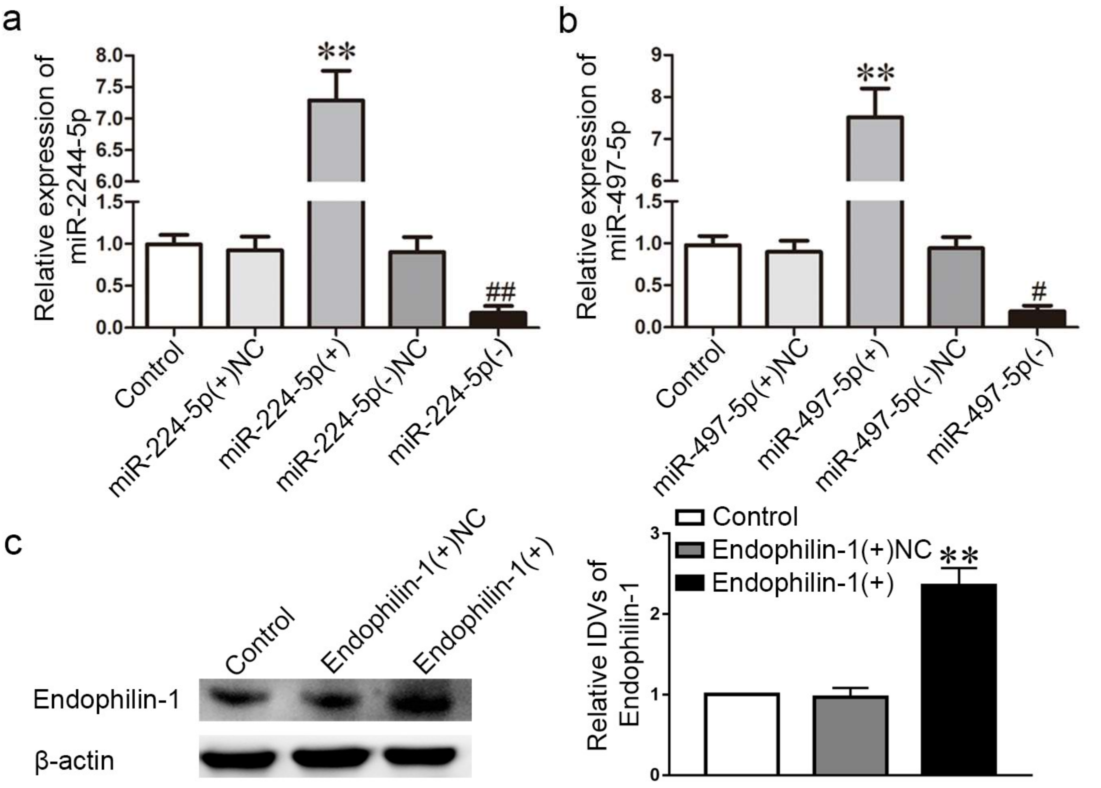

Figure S3. Transfection efficiency of LINC00094, miR-224-5p/ miR-497-5p and Endophilin-1. **(a)** Relative expression of miR-224-5p in cells transfected agomir-224-5p or antagomir-224-5p. Data represent mean ± s.d. (n=3, each). $^{**}P <$ 0.01 vs. miR-224-5p (+) NC group, $^{\#\#}P < 0.01$ vs. miR-224-5p (-) NC group. **(b)** Relative expression of miR-497-5p in cells transfected agomir-497-5p or antagomir-497-5p. Data represent mean ± s.d. (n=3, each). $^{**}P < 0.01$ vs. miR-497-5p (+) NC group, $^{\#}P < 0.05$ vs. miR-497-5p (-) NC group. **(c)** Relative expression of Endophilin-1 in cells transfected pIRES2-EGFP-Endophilin-1-CDS. Data represent mean ± s.d. (n=3, each). $^{**}P < 0.01$ vs. Endophilin-1 (+) NC group.
