# Supplementary material for: The role of LINC00094/miR‐224‐5p (miR‐497‐5p)/Endophilin‐1 axis in Memantine mediated protective effects on blood‐brain barrier in AD microenvironment
Source: J Cell Mol Med. 2019 Feb 22;23(5):3280–92. doi: 10.1111/jcmm.14214 (PMC6484416; doi:10.1111/jcmm.14214)
Supplement: Supplementary file 2 [file JCMM-23-3280-s002.pdf]

The Role of LINC00094/ miR-224-5p (miR-497-5p)/ Endophilin-1 Pathway in Memantine Regulating the Permeability of Blood-Brain Barrier

Lu Zhu[a], Meiqing Lin[a], Jun Ma[b, c], Wenjing Liu[d], Lili Gao[a], Shanshan Wei[a], Yixue Xue[b,c,], Xiuli Shang[a, *]

Table S1. Primer and probes used for RT-qPCR

Primer or Probe	Gene	Sequence(5'->3') or Assay ID
Primer	LINC00094	F:ATGGAAATGAGGCCATCTTG
		R:AAGTGTGGTCCTGGGTTCAG
	GAPDH	F:CCCATCACCATCTTCCAGGAG
		R:GTTGTCATGGATGACCTTGGC
Probe	miR-224-5p	002099(Applied biosystems)
	miR-497-5p	001043(Applied biosystems)
	Endophilin-1	Hs00182352_ml(Applied biosystems)
	U6	001973(Applied biosystems)
	GAPDH	Hs03929097_gl(Applied biosystems)

Table S2. Sequences of shRNA template

Gene		Sequence(5'->3')
LINC00094	Sence	CACCGCAGGTGTCTCTTGTCAAAGCTTCAAG
		AGAGCTTTGACAAGAGACACCTGCTTTTTTG
	Antisence	GATCCAAAAAAGCAGGTGTCTCTTGTCAAAG
		CTCTCTTGAAGCTTTGACAAGAGACACCTGC
NC	Sence	CACCGTTCTCCGAACGTGTCACGTCAAGA
		GATTACGTGACACGTTCGGAGAATTTTTTG
	Antisence	GATCCAAAAAAGTTCTCCGAACGTGTCACG
		TAATCTCTTGACGTGACACGTTCGGAGAAC
